# Secondary Pharmacotherapeutic Prevention among German Primary Care Patients with Peripheral Arterial Disease

**DOI:** 10.1155/2011/316496

**Published:** 2011-06-26

**Authors:** Uwe Müller-Bühl, Gunter Laux, Joachim Szecsenyi

**Affiliations:** Department of General Practice and Health Services Research, University Hospital, University of Heidelberg, Voßstrasse 2, Geb. 37, 69115 Heidelberg, Germany

## Abstract

*Background*. The aim of the study was to determine the secondary preventive medical supply of patients with peripheral arterial disease (PAD) in German primary care. *Methods and Results*. A population-based case control study was conducted using electronic medical records of patients extracted from the CONTENT primary care database of Heidelberg, Germany, between April 2007 and March 2010. The prescription rates of cardiovascular medication among symptomatic PAD patients were analysed by means of the ATC classification and compared with those of patients with cardiovascular disease (CVD). 479 cases with PAD and 958 sex- and age-matched control CVD patients were identified. PAD patients showed significantly lower prescription rates for cardiac agents (21.7% versus 37%), **β**-blockers (50.1% versus. 66.2%), and lipid-lowering agents (50.3% versus 55.9%) compared to CVD patients. In contrast, significantly more prescriptions of antidiabetic agents (28.2% versus 20.3%), particularly insulin and analogues (12.5% versus 8%), and calcium channel blockers (29.2% versus 24.3%) were found in PAD patients. Low-dose aspirin use among both PAD and CVD patients was underestimated, as it is available without a prescription. *Conclusions*. Optimal pharmacotherapeutical care of patients with PAD requires more intensive cardioprotective medication in primary care settings.

## 1. Introduction

Pharmacotherapy in patients with peripheral arterial disease (PAD) aims to prevent progression of atherosclerotic disease, to reduce risk of global cardiovascular events and to improve walking capacity. Since PAD is a manifestation of systemic atherosclerosis in the lower extremities, its pharmacotherapeutic goals are nearly identical to those of the cerebrovascular disease and cardiovascular disease (CVD). Smoking cessation, exercise, and an aggressive pharmacological risk factor modification, including diabetes, hypertension, lipid abnormalities, and life-long antiplatelet therapy, represent the cornerstones of treatment. The therapeutical recommendations based on solid research are included in current national and international PAD guidelines. Despite strict guideline directives, the PAD is still considered today as undertreated in many countries [1–4]. In particular, an underuse of PAD treatment has been documented compared to treatment of CVD patients [5–8]. 

In Germany, high prescription rates for secondary preventive medication in PAD patients primarily treated by vascular specialists were recently reported [9]. The objectives of the study were to examine whether an accordingly intensive pharmacological PAD patient treatment is also realized in primary care, particularly when compared with those of CVD patients.

## 2. Methods 

### 2.1. Data Source

The CONTENT (CONTinuous morbidity registration Epidemiologic NETwork) database is a national primary care research project which is created and maintained by the Department of General Practice and Health Services Research of the University Hospital Heidelberg. The structure and core elements of this datapool have been described elsewhere [10]. In brief, based on the International Classification of Primary Care (ICPC-2) [11], all electronic, routinely documented elements of doctor-patient encounters in 34 primary care settings in Southwest Germany are registered continuously and are available for analysis of specific problem areas. The study protocol was approved by the ethics committee of the University of Heidelberg (registration number 442/2005).

### 2.2. Study Sample

The electronic patient records of 105,026 primary care patients across a three-year period (April 1, 2007–March 31, 2010) were screened in an anonymous manner. Patients with PAD were identified using the International Classification of Disease (ICD-10) code I73.9. CVD patients were selected from the ICD-10 codes I20 and I25. This method allows extraction of all PAD/CVD patients who exhibit ensured and coded diagnoses in their electronic patients record. Thus not only patients who had become conspicuous through their symptoms and were definitively diagnostically clarified were detected, but also those who have become asymptomatic subsequent to PTA/PTCA treatment. However, we were not able to extract, for example, information about the number of therapeutic interventional or surgical procedures and their possible medical success or consequences. Asymptomatic patients, and thus the majority of PAD patients, could not be identified because ABI measurement was not available as a PAD selection criterion. Furthermore, patients presenting with atypical claudication symptoms, for example, patients with superimposing coincident neuropathy or osteoarthrosis, could not be identified. This is traced to a nonexistent ICPC code or ICD-10 code for the Doppler sonographic ABI measurement. Subjects presented coincidentally with PAD code and CVD code were excluded from the analysis. Moreover, only patients with continuous contact to their family practitioner and properly documented prescriptions were included. In order to obtain comparable cohorts, we identified determinants for PAD on the basis of binary logistic regression. Based on these variables, matching was performed with the Propensity Score (PS) method proposed by Rosenbaum and Rubin [12]. The package “Matching of R” (Version 2.12.0) [13] was used for the matching procedure. For evaluation of the matching results the “MatchBalance” function of this package was applied, since this function not only examines differences of means, but also accounts for potential differences across the entire distribution [14].

Sociodemographic variables (e.g., education or profession) were not available for the analysis. The “chronic conditions” were defined on the basis of ICPC codes according to the concept of O'Halloran et al. [15] that regards diagnoses as well as chronic symptoms and complaints. 

All prescriptions corresponding to the PAD code and the CVD code were analysed by means of the Anatomical Therapeutic Chemical Classification (ATC). The ATC system divides the drugs into different groups according to the organ or system on which they act and according to their chemical, pharmacological, and therapeutic properties. Drugs are classified in 14 main groups (first level) and spread out into therapeutic/pharmacological subgroups down to the plain chemical substances (fifth level) [16].

### 2.3. Statistics

In order to assess potential differences in pharmacotherapy between PAD and CVD patients in matters of well-defined ATC subgroups, contingency comparisons were performed using Fisher's Exact Test. A significance level of 0.05 was defined for hypothesis testing. Statistical analyses were performed with “R” (Version 2.12.0).

## 3. Results

479 PAD patients and 1,972 CVD patients met the inclusion criteria. The matched-pair procedure resulted in 958 corresponding CVD patients, that is, a PAD/CVD patient ratio of 1 : 2. Binary logistic regression defined patients' age, gender, practice setting, and type of health insurance (statutory versus private) as significant determinants for the analysis (Table 1). The matching procedure resulted in an exact match for patients' gender and practice setting for the entire PAD group. The percentage of patients with private health insurance was slightly but not significantly lower in the PAD cohort (7.1% PAD versus 9.0% CVD). There was no significant difference in average age at the end of the observation period for PAD patients and CVD patients. The “MatchBalance” function indicated that patients' age in both cohorts was distributed very similarly. There was no difference between PAD and CVD patients concerning the number of “chronic conditions”. As was expected, the studied patient cohorts were of markedly older age compared to the total register sample (Table 2).

Table 3 shows that the total number of prescribed drugs did not differ between the PAD and CVD cohorts. However, compared to the PAD patients, the CVD patients received significantly more drugs of the ATC level 1 group C “cardiovascular system”. The more detailed ATC level 2 subgroup analysis suggested that this distinction is primarily based on higher prescription rates for cardiac therapy agents, *β*-blockers, and lipid-lowering agents. In contrast, PAD patients presented significantly more frequent prescriptions for antidiabetic agents than CVD patients, particularly more insulin and analogues and more calcium channel blockers. 

The analysis of ATC drug levels 4 and 5 showed that the difference between prescriptions of lipid-lowering agents is mainly caused by higher HMG-CoA reductase inhibitor nominations among CVD patients, in particular simvastatin prescriptions. The ATC groups level 5 of antithrombotic agents suggested equal prescription rates for aspirin and clopidogrel between the CVD and PAD cohorts. Remarkably, no cilostazol prescriptions were registered.

## 4. Discussion

In the present study, we attempt to depict the secondary medical prevention among symptomatic patients with PAD compared to CVD patients in German primary care. The analysis of the GP's prescription dataset suggested no substantial difference in the total number of prescribed drugs between PAD and CVD patients. This concerned all medications except cardiovascular drugs, which more frequently emerged in the prescriptions for cardiac patients than in those of patients with peripheral vascular disorder. This distinction is based on significantly more dispensations of specific cardiac agents for CVD patients, for example, glycosides, antiarrhythmics, or nitrates, and particularly on more prescribed *β*-blockers. The latter disparity accords with studies conducted in the U.S. and Europe, which reported marked differences for treatment with *β*-blockers between the two studied populations [1, 6, 17]. This may not only be related to the fact that CVD management is mainly influenced by specialists, whereas PAD is largely managed in primary care. There is also an incomprehensible restraint of some physicians to administer *β*-blockers among PAD patients, although *β*-blocker therapy was proven as being nondetrimental to walking capacity [18] and is even related to a significant independent decrease in new coronary events [19]. 

Little is known about the secondary preventive effects of other classes of antihypertensive drugs in the presence of PAD [20]. In our study, 67% of both the PAD and CVD patients obtained ACE inhibitors/AT-II receptor antagonists. Our data are consistent with results of other inpatient and outpatient studies [6, 7, 9, 17]. Notably, PAD patients received significantly more calcium channel blockers than patients with CVD, perhaps as a consequence of GPs' contemplating the peripheral vasodilatation effect of these agents. The data of the German REACH study [6] demonstrated a similar but not significantly higher administration of calcium-antagonists in PAD patients than in patients with CVD (31% versus 27%).

As an effective secondary-preventive measure, the PAD guidelines recommend lifelong treatment of symptomatic patients with statins [21]. The 4S Study of 4,444 patients with known cardiovascular disease revealed that use of simvastatin reduced episodes of new or worsening intermittent claudication [22]. Moreover, the Heart Protection Study demonstrated that statins reduced coronary death in PAD patients irrespective of their initial cholesterol value [23]. In our study, only half of the PAD patients received an appropriate lipid-lowering therapy. In accordance with the literature this confirms an undertreatment of PAD patients with lipid-lowering agents in primary care, in particular, in view of the advanced stage of the disease [7, 9, 24]. 

Despite PAD guideline recommendations for secondary prevention, the effect of aspirin in this population is not well established. A meta-analysis of eighteen prospective randomized trials involving 5269 participants resulted in a demand for additional randomized controlled trials of aspirin therapy to establish the real benefit and bleeding risks in PAD [25]. Nevertheless, in numerous more recent studies, patients with atherothrombotic diseases are univocally considered as aspirin underused [1, 7, 17, 26]. However, the findings of our study cannot contribute a substantial result to this issue. The comparably low aspirin prescription rates of 43% in both PAD and CVD cohorts do not reflect the real intake of low-dose aspirin by these patients, because in German pharmacies aspirin compounds are obtainable as over-the-counter medication being even cheaper than prescribed aspirin copayment. 

The findings of our study suggest an inadequate preventive medical therapy in patients with PAD in German family practice settings as had already been demonstrated in other studies [1–7, 17, 24, 25]. Taking into account that the study sample consists of PAD patients suffering from an advanced stage of the disease, an overestimation of the general treatment prevalence in PAD/CVD patients can be assumed. The real cardiovascular drug supply of patients with PAD would probably be less intensive if asymptomatic PAD patients were additionally included in the statistical analysis. The reason for the underuse of preventive cardiovascular medication in patients with atherosclerotic disease remains speculative. Besides a deficient realization of guideline recommendations, a general lacking awareness among physicians of the PAD patients cardiovascular hazard could be supposed, tending to result in neglect of appropriate medication [27, 28].

We are aware of the limitations inherent to any study extracting data from electronic patient records. The main weakness of our study is the data collection by means of ICD/ICDC coded diagnoses which disregards asymptomatic patients with PAD/CVD and is more selective with regard to the advanced stages of the atherosclerotic disease. Furthermore, a potential selection bias must be admitted because a GP's participation in the CONTENT registry is voluntary and not by random selection. In view of the considerable number of participating practices and analyzed patients, it can nevertheless be assumed that our study findings give at least a realistic insight into the drug treatment of PAD patients in primary care. We should also underline that with the ATC classification we only analysed plain chemical substances and not compound medicines, and for this reason a slight underestimation of true agent use cannot be ruled out. Altogether, we believe the key information of our study findings is not substantially affected by these limitations, especially as a possible bias involves a systematic error for both the PAD and CVD cohorts.

## 5. Conclusion

The findings of the present study reflect an overall picture of secondary prevention drug management in patients with PAD in German primary care. In accordance with the international literature, the treatment guidelines have not been sufficiently enough translated into family practice settings. The analysed PAD patient sample was less intensively treated with *β*-blockers, ACE inhibitors, and lipid-lowering agents than comparable CVD patients. As a consequence, serious considerations should be taken to implement the PAD therapy guidelines recommendations more effectively in practice.

## Figures and Tables

**Table 1:** Study patient characteristics.

	PAD patients	CVD patients	*P*-value
Number	479	958	n.s.
Age			
mean (SD)	72.6 (12.0)	72.9 (11.2)	n.s.
Gender			
males (%)	56.4	56.4	n.s.
females (%)	43.6	43.6	n.s.
Number “chronic conditions”			
mean (SD)	4.1 (3.7)	4.2 (3.6)	n.s.
Number encounters (per mille)	38.8 (28.3)	37.2 (29.0)	n.s.
Practice profile^‡^	100%		

^‡^corresponding PAD patients and CAD patients were matched within the same practice setting without exception.

**Table 2:** Prevalence of PAD within different age groups of the entire study population.

Age group (years)	3-year contact group		PAD	
	*n*	%	*n*	%
0–10	7700	7.33	0	0.00
11–20	10443	9.94	0	0.00
21–30	12930	12.31	1	0.21
31–40	12933	12.31	3	0.63
41–50	17533	16.69	15	3.13
51–60	13893	13.23	68	14.20
61–70	11588	11.03	125	26.10
71–80	10212	9.72	155	32.36
81–90	6379	6.07	98	20.46
>90	1415	1.35	14	2.92
Total	**105 026**	**100.0**	**479**	**100.0**

**Table 3:** Number of selected pharmacotherapeutical prescriptions in a 3-year study group of PAD and CVD patients by means of ATC classification.

	PAD (*n* = 479)	CVD (*n* = 958)	*P* value
Total number, mean (SD)	46.9 ± 39.9	42.8 ± 37.7	n.s.
ATC groups level 1			
A alimentary tract and metabolism	322 (67.2%)	618 (64.5%)	n.s.
B blood and blood forming organs	318 (66.4%)	615 (64.2%)	n.s.
C cardiovascular system	405 (84.6%)	849 (88.6%)	<.05
ATC groups level 2			
A 10 antidiabetic agents	135 (28.2%)	194 (20.3%)	<.001
B 01 antithrombotic agents	303 (63.3%)	584 (61.0%)	n.s.
C 01 cardiac therapy agents	101 (21.1%)	354 (37.0%)	<.0001
C 07 *β*-blockers	240 (50.1%)	635 (66.2%)	<.0001
C 08 calcium channel blockers	140 (29.2%)	233 (24.3%)	<.05
C 09 renin-angiotensin system agents	322 (67.2%)	656 (68.5%)	n.s.
C 10 antihyperlipidemics	241 (50.3%)	536 (55.9%)	<.05
ATC groups level 3 + 4			
A 10A insulins and analogues	60 (12.5%)	77 (8.0%)	<.05
A 10B oral blood glucose lowering agents	96 (20.0%)	156 (16.3%)	n.s.
B 01AA Vitamin K antagonists	56 (11.7%)	124 (12.9%)	n.s.
B 01AB heparin and derivates	50 (10.4%)	97 (10.1%)	n.s.
B 01AC platelet aggregation inhibitors	254 (53.0%)	481 (50.2%)	n.s.
C 09AA ACE inhibitors, plain	198 (41.3%)	439 (45.8%)	n.s.
C 10AA HMG CoA reductase inhibitors	222 (46.3%)	504 (52.6%)	<.05
ATC groups level 5			
B 01AC04 clopidogrel	101 (21.1%)	186 (19.4%)	n.s.
B 01AC06 acetylsalicylic acid	206 (43.0%)	420 (43.8%)	n.s.
B 01AC23 cilostazol	0 (0.0%)	0 (0.0%)	n.s.
C 09AA05 ramipril	139 (29.0%)	310 (32.4%)	n.s.
C 10AA01 simvastatin	195 (40.7%)	443 (46.2%)	<.05
